# Effect of Quercetin in the 1-Methyl-4-phenyl-1, 2, 3, 6-tetrahydropyridine-Induced Mouse Model of Parkinson's Disease

**DOI:** 10.1155/2012/928643

**Published:** 2012-02-09

**Authors:** Chuanfeng Lv, Tie Hong, Zhen Yang, Yu Zhang, Lu Wang, Man Dong, Jing Zhao, Jiaye Mu, Yixiao Meng

**Affiliations:** ^1^Department of Pharmacology, School of Pharmacy, Jilin University, Changchun 130021, China; ^2^Institute of Changbai Mountain Natural Resources, Jilin Academy of Chinese Medicine Sciences, Changchun 130012, China; ^3^College of Pharmacy, Yanbian University, Yanji 133002, China

## Abstract

In this paper, the protective effect of the bioflavonoid quercetin on behaviors, antioxidases, and neurotransmitters in 1-methyl-4-phenyl-1, 2, 3, 6-tetrahydropyridine-(MPTP-) induced Parkinson's disease (PD) was investigated. Quercetin treatment (50 mg/kg, 100 mg/kg and 200 mg/kg body weight) was orally administered for 14 consecutive days. The results show that quercetin treatment markedly improves the motor balance and coordination of MPTP-treated mice. Significant increases were observed in the activities of glutathione peroxidase (GPx), superoxide dismutase (SOD), and Na^+^, K^+^-ATPase, AchE, the content of dopamine (DA) in the quercetin plus MPTP groups compared to those in the MPTP group. Significant reduction the 4-hydroxy-2-nonenal (4-HNE) immunoreactivity in striatum of brains was observed in the quercetin plus MPTP groups in comparison to the MPTP group. Taken together, we propose that quercetin has shown antiparkinsonian properties in our studies. More work is needed to explore detailed mechanisms of action.

## 1. Introduction

Parkinson's disease (PD), which is a type of regressive disease of the central nervous system, is the second most common disorder of the aging brain after Alzheimer's disease. The clinical manifestations are static tremors, myotonia, bradykinesia, and poor balance. The main drug families that are useful for treating motor symptoms are levodopa, dopamine agonists, and MAO-B inhibitors [1].

PD models are divided into two broad categories: genetic and toxic [2, 3]. Most of the data that addresses the effects of oxidative stress have been generated using toxic models such as those produced by 1-methyl-4-phenyl-1, 2, 3, 6-hydroxydopamine (MPTP), which damages the nigrostriatal dopaminergic system. 

The antioxidative activity of quercetin, which is a bioflavonoid, has been studied [4]. Recent studies have shown that quercetin crosses the blood-brain barrier (BBB) [5] and protects HT-22 cells by prohibiting the formation of reactive oxygen species (ROS), which is generated by glutamic acid-induced oxidation toxicity and lipid peroxidation [6]. 

In the present study, we investigated the neuroprotective effects of quercetin to modify glutathione peroxidase (GPx), superoxide dismutase (SOD), Na^+^, K^+^-ATPase, immunoreactivity of 4-hydroxy-2-nonenal (4-HNE), acetylcholinesterase (AChE) activities, and the level of dopamine (DA) in the brain tissue in the MPTP-induced mouse model of Parkinson's disease.

## 2. Materials and Methods

### 2.1. Animals

Specific pathogen-free adult male C57BL/6 mice (25 ± 2 g, body weight; 2–2.5 months old) were housed in standard cages with wood shavings. Ten animals per cage were kept in a room with a carefully controlled ambient temperature (25°C) and artificially illumination (12 hours of light from 8:00 AM to 8:00 PM). All experiments were performed under the Guidelines of the Experimental Laboratory Animal Committee of the Jilin Province.

### 2.2. Drugs

Quercetin (98%) and MPTP were purchased from Sigma-Aldrich (St. Louis, MO, USA). All of the other chemicals were of analytical grade and obtained from standard commercial suppliers. MPTP was dissolved in 0.9% physiological saline, and quercetin was resuspended in distilled water.

### 2.3. Animal Grouping and Treatment 

The mice were divided into 5 groups (10 mice in each group): the control group, the MPTP group (MPTP-treatment group), the low-dose group (quercetin 50 mg/kg body weight plus MPTP), the middle-dose group (quercetin 100 mg/kg body weight plus MPTP), and the high-dose group (quercetin 200 mg/kg body weight plus MPTP). All quercetin plus MPTP groups were orally administered the indicated concentration of quercetin every 24 h for 14 consecutive days. To evaluate the effects of quercetin in the PD mouse model, MPTP was intraperitoneally injected with five consecutive injections at a dose of 30 mg/kg every 24 h from day 10 to day 14 starting at 1 h after the oral administration of quercetin. An equal volume of saline instead of MPTP was injected into the mice in the control group.

### 2.4. Motor Behavior Analysis with the Rotarod Test

An accelerating rotarod test was used to measure motor balance and coordination in mice as described previously by L'Episcopo et al. [7]. The rotarod cylinder was accelerated from 4 to 40 rpm within 5 minutes, and the cutoff time was 300 seconds. The total time that each mouse remained on the rod was automatically recorded by a trip switch under the floor of each rotating drum that was activated by the animal's fall. The average time (fall latency) for three trials was determined.

### 2.5. Tissue Sample

The mice were sacrificed after being anesthetized with pentobarbital sodium. The brain tissue was isolate removed and divided equably into two parts. 

One part of the brain tissues was immediately homogenized in cold 10 mM Tris-HCl, pH 7.5 (1/10 w/v), with 10 up-and-down strokes at approximately 12,000 rpm in a Teflon-glass homogenizer. The homogenates were centrifuged at 3000 ×g for 10 min to yield a clear supernatant fraction as the sample that was used for measuring the activity of GPx, SOD, Na^+^, K^+^-ATPase, and AChE. 

The other part of the brains was transferred into a 1.5 mL plastic vial, then weighed and homogenized in iced-cold HClO_4 _(0.4 M) using an ultrasonicator. After storage for 1 h in ice, the homogenates were centrifuged at 12,000 ×g for 15 min at 4°C. The supernatant was incubated with a mixed buffer (20 mM sodium citrate, 300 mM K_2_HPO_4_, 2 mM sodium ethylenediaminetetraacetic acid (Na_2_EDTA)) at the ratio (v/v) of 1 : 2 for 1 h on ice and centrifuged at 12,000 ×g for 15 min at 4°C. The supernatant was collected and filtered through a 0.22 mm filter and was subsequently analyzed the level of the DA [8].

### 2.6. Biochemical Analysis

The GPx activity was determined in accordance with a previously described method [9] with minor modifications. The enzymatic activity of GPx was represented as unit/mg protein, where 1 unit of GPx activity was defined as 1 *μ*M GSH that was depleted per minute. 

The SOD activity was measured using the method of Ohkawa et al. [10] to evaluate the ability of the xanthine-xanthine oxidase system to inhibit the oxidation of oxymine. 

Na^+^, K^+^-ATPase activity was determined using method that has been described by Wyse et al. [11]. The reaction mixture for the Na^+^, K^+^-ATPase activity assay contained the following: 3 mM MgCl_2_, 125 mM NaCl, 20 mM KCl, and 50 mM Tris-HCl (pH 7.4) in a final volume of 500 *μ*L. The reaction was initiated by the addition of ATP to a final concentration of 3.0 mM. The control samples were obtained under the same conditions with the addition of 0.1 mM ouabain. The samples were incubated at 37°C for 30 min, and the incubation was stopped by adding trichloroacetic acid solution (10% TCA) with 10 mM HgCl_2_. Na^+^, K^+^-ATPase activity was calculated based on the difference between the two assays (with ouabain/without ouabain). The specific activity of the enzyme was expressed as mol Pi that was released per hour per mg of protein (mol Pi/hour/mg protein). 

The activity of acetylcholinesterase (AchE) was estimated using the method of Ellman et al. [12]. The samples were homogenized in buffer medium I (0.32 M sucrose, 5 mM Tris-HCl and 0.1 mM EDTA) (1 : 10; w/v) and centrifuged at 2400 ×g for 10 min to obtain the low-speed supernatant. The homogenate (100 *μ*L) was incubated in 1 mL of a solution containing 10 mM 5,5-ditiobis (2-dinitrobenzoic) acid (DTNB) (dissolved in potassium phosphate buffer pH 7.0) and 100 mM potassium phosphate buffer (pH 7.5) as well as 700 *μ*L water for 2 min at 25°C. Afterwards, 200 *μ*L of acetylthiocholine (8 mM, substrate) was added to the tube test. The activity of AchE was spectrofluorometrically measured at 412 nm and expressed as hydrolyzed Ach/min/mg protein. 

The protein concentration was measured using the Bradford method [13] with bovine serum albumin as the standard. 

The level of DA was determined using HPLC, which was equipped with an electrochemical (EC) detector that was used for quantification [8]. The level of DA was determined using standard curves as references. The data were expressed as ng/g tissue weight.

### 2.7. Immunohistochemistry

Immunohistochemical method was taken in another batch of mice which were treated as the “animal grouping and treatment.” The brains were quickly removed and postfixed for 2 days with paraformaldehyde. Immunohistochemical studies were performed on paraffin-embedded midbrain sections. The 30 *μ*m thick transverse sections were deparaffinized with xylene and refixed with Bouin's solution for 20 min. For inhibition of endogenous peroxidase, the sections were incubated with 0.3% H_2_O_2_ in methanol for 30 min. After rinsing in 10 mM phosphatebuffered saline (PBS), the sections were incubated with normal goat serum (Dako, diluted to 1 : 10) to inhibit nonspecific binding of the antibodies. After incubation with the polyclonal anti-4-hydroxy-2-nonenal (HNE) antibody (1 : 400, Alpha Diagnostic International, San Antonio, TX, USA) at 4°C overnight, the sections were treated with biotinylated secondary antibody for 1 h at 37°C, then with streptavidin-peroxidase for 1 h. Subsequently the sections were incubated with 3, 4-diaminobenzidine. The results were analyzed by counting the numbers of positive cells at 400x magnifications on a microscope (Eclipse 80i, Nikon Corp., Japan). The average number of positive cells was used to represent cell density.

### 2.8. Statistics

All of the data were expressed as the mean ± SE. The statistical significance of differences that were detected in each parameter among the groups was evaluated using one-way analysis of variance (ANOVA) followed by Fisher's protected least significant difference (PLSD) comparison tests for *post hoc t*-tests.

## 3. Results

### 3.1. The Results of the Rotarod Test

**Figure 1:**
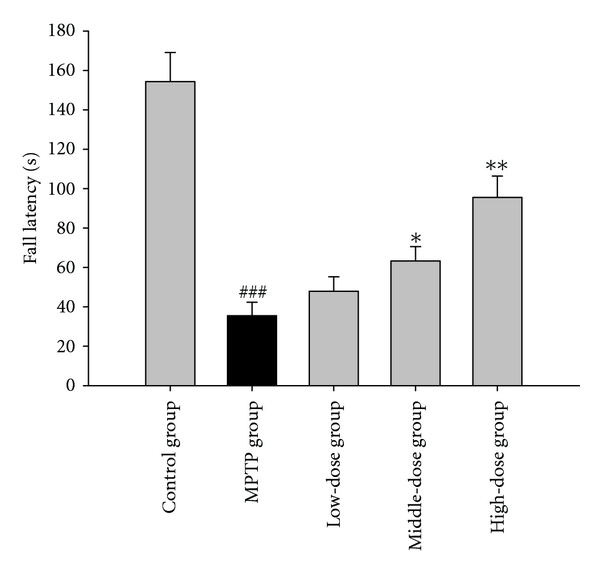
The effect of quercetin on the fall latency of rotarod test in a mice model of Parkinson's disease (PD) induced by MPTP. Data were expressed as mean ± SE for 10 mice in each group. ^###^: *P* < 0.001 as compared to the control group; *: *P* < 0.05 as compared to MPTP group; **: *P* < 0.01 as compared to MPTP group.

In the rotarod test, the fall latency of the MPTP group was significantly shorter than that of the control group (*P* < 0.001). Compared with the MPTP group, the quercetin low-dose group showed no significant change whereas the middle- and high-dose groups exhibited significantly longer fall latencies (*P* < 0.05 and *P* < 0.01, resp.). The results are shown in Figure 1. 

### 3.2. The Effect on Antioxidases

**Figure 2:**
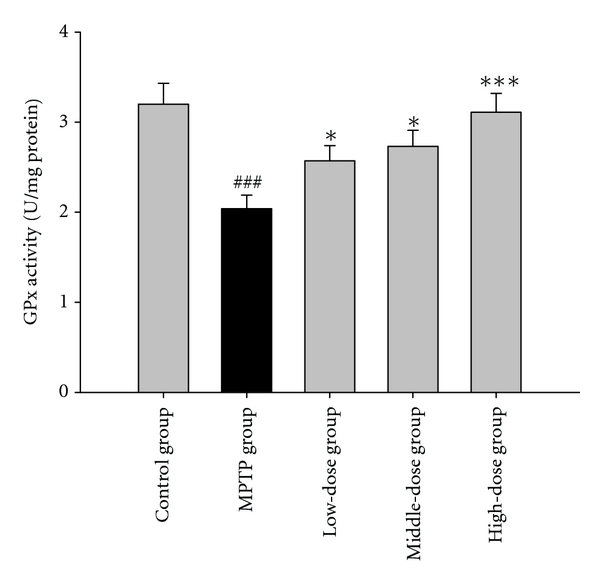
The effects of quercetin on the activity of glutathione peroxidase in a mice model of Parkinson's disease (PD) induced by MPTP. Data were expressed as mean ± SE for 10 mice in each group.^ ###^: *P* < 0.001 as compared to the control group; *: *P* < 0.05 as compared to MPTP group; ***: *P* < 0.001 as compared to MPTP group.

**Figure 3:**
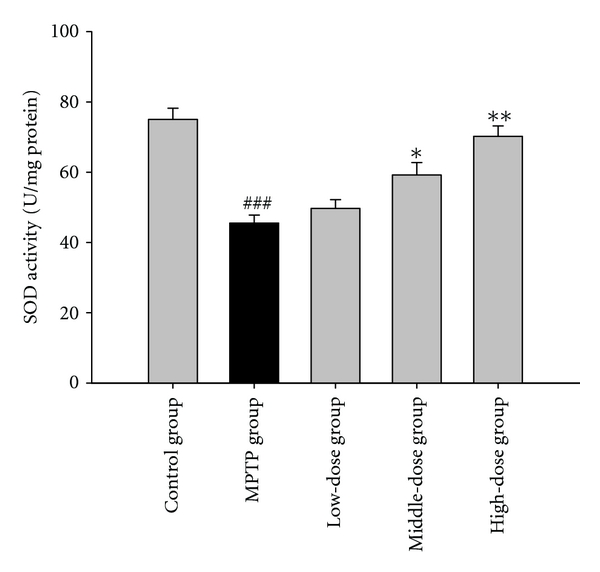
The effects of quercetin on the activity of superoxide dismutase in a mice model of Parkinson's disease (PD) induced by MPTP. Data were expressed as mean ± SE for 10 mice in each group. ^###^: *P* < 0.001 as compared to the control group; *: *P* < 0.05 as compared to MPTP group; **: *P* < 0.01 as compared to MPTP group.

**Figure 4:**
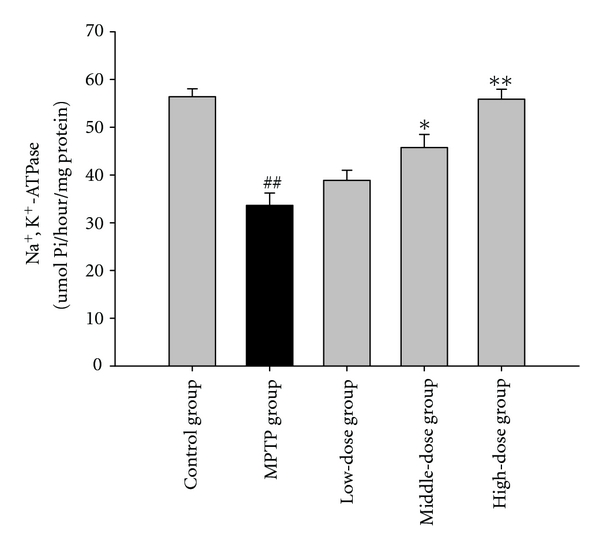
The effects of quercetin on the activity of Na^+^, K^+^-ATPase in a mice model of Parkinson's disease (PD) induced by MPTP. Data were expressed as mean ± SE for 10 mice in each group. ^##^: *P* < 0.01 as compared to the control group; *: *P* < 0.05 as compared to MPTP group; **: *P* < 0.01 as compared to MPTP group.

MPTP-treated mice displayed perturbations in the activities of GPx, SOD, and Na^+^, K^+^-ATPase. Pretreatment with quercetin augmented the activities of GPx, SOD, and Na^+^, K^+^-ATPase in the mouse brain tissue. The results are shown in Figures 2, 3 and 4.

### 3.3. The Result of Immunohistochemistry

**Figure 5:**
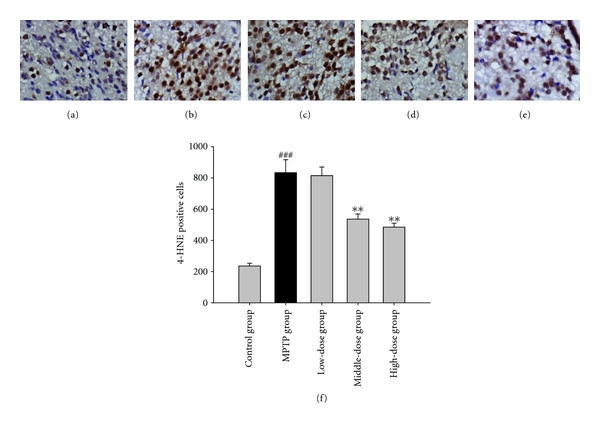
Immunostaining of striatum with 4-HNE in a mice model of Parkinson's disease (PD) induced by MPTP. Staining was present within cell bodies. ^###^: *P* < 0.001 as compared to the control group: **: *P* < 0.01 as compared to MPTP group. (a) In control group, there was a few of 4-HNE immunoreactivity. (b) MPTP group: compared with control group, MPTP markedly increased the immunoreactivity of 4-HNE in mice striatum. (c) Low-dose group: no difference from MPTP group. (d) Middle-dose group: 4-HNE immunoreactivity are significantly reduced in striatum of brains in comparison to the MPTP group. (e) High-dose group: 4-HNE immunoreactivity are significantly reduced in striatum of brains in comparison to the MPTP group.

Immunoreactivity of 4-HNE was markedly increased in the striatum of the mice PD brain compared to the control group brain that displayed weak immunoreactivity of 4-HNE. We assessed the relationship of the antioxidant effect of quercetin in PD mice through phenotypic observation along with 4-HNE using microscopy. Quercetin (100, 200 mg/kg) reduced the immunoreactivity of 4-HNE in the striatal neurons of C57/BL mice. All results shown in Figure 5. 

### 3.4. The Effect on AchE

**Figure 6:**
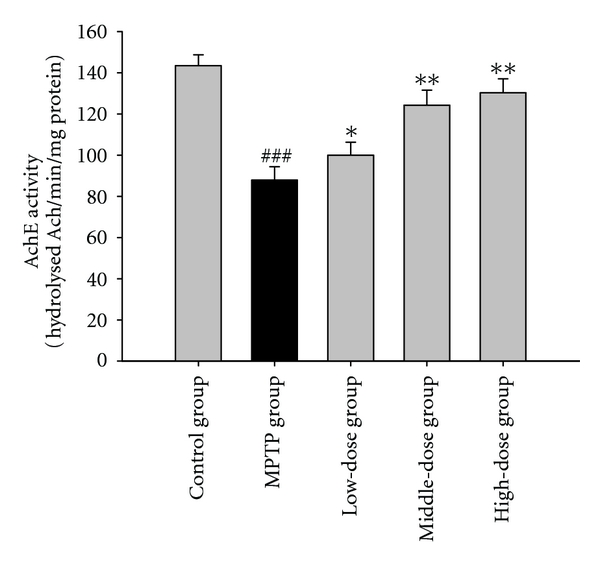
The effects of quercetin on the activity of acetylcholinesterase in a mice model of Parkinson's disease (PD) induced by MPTP. Data were expressed as mean ± SE for 10 mice in each group. ^###^: *P* < 0.001 as compared to the control group; *: *P* < 0.05 as compared to MPTP group; **: *P* < 0.01 as compared to MPTP group.

We analyzed the effects of quercetin on the activity of AchE in the brain tissues. AchE was lower in the MPTP group than in the control group. AchE in administration of quercetin groups showed higher than that MPTP group. The results were showed in Figure 6.

### 3.5. The Effect of the Level of DA

**Figure 7:**
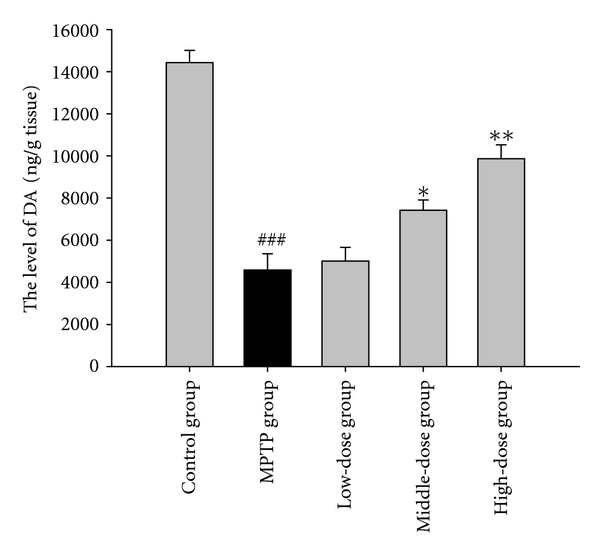
The effects of quercetin on the level of dopamine in a mice model of Parkinson's disease (PD) induced by MPTP. Data were expressed as mean ± SE for 10 mice in each group. ^###^: *P* < 0.001 as compared to the control group; *: *P* < 0.05 as compared to MPTP group; **: *P* < 0.01 as compared to MPTP group.

The effects of quercetin on the levels of DA in the brain are shown in Figure 7. The brain levels of DA decreased in the MPTP group compared to the control group (*P* < 0.01). Compared with the MPTP group, the quercetin pretreatment groups attenuated the MPTP-induced DA depletion.

## 4. Discussion

MPTP is metabolized into the toxic cation 1-methyl-4-phenylpyridinium (MPP+) by the enzyme MAO-B in glial cells. MPP+ primarily kills dopamine-producing neurons in the part of the brain that is called the pars compacta of the substantia nigra and interferes with a component of mitochondrial metabolism, which induces cell death and causes the buildup of free radicals and toxic molecules [14]. 

The rotarod test is designed to evaluate the motor coordination and balance of the mouse by forcing the animal to run. In the present study, the results of the rotarod test reveal that the quercetin concentrations at the middle (100 mg/kg) or high (200 mg/kg) doses enhance the motor activity of MPTP-treatment mice. This finding shows that quercetin plays an important role in the developmental processes of the internal system regulating the mouse motor behavior and suggests that quercetin improves the balance of MPTP-treatment mice. 

Recent research has suggested that the brain may efficiently metabolize superoxide but may have difficulties regarding hydrogen peroxide elimination [15]. In this study, the MPTP group showed lower GPx and SOD activities. These results indicate that MPTP reduces the elimination of hydrogen peroxide and free radicals in the brain. Quercetin pretreatment in the MPTP-induced group showed increased GPx and SOD activities. These results suggest that the elimination of superoxide anion was enhanced by quercetin. Hydrogen peroxide, which is generated during superoxide dismutation, was sufficiently removed by GPx after the pretreatment with quercetin. 4-HNE is a major membrane lipid peroxidation product [16]. Additionally, we analyzed the effect of quercetin on the level of 4-HNE in striatal neurons. As expected, quercetin (100, 200 mg/kg body weight) reduced the 4-HNE immunoreactivity in neurons of PD model mice. It shows that quercetin could decrease the product of lipid peroxidation. These results indicate the mechanism of quercetin neuroprotection effects. 

The Na^+^, K^+^-ATPase establishes the ionic concentration balance that maintains the cell's potential. Recent research has suggested that the Na^+^, K^+^-ATPase is susceptible to free radical-induced damage [17]. In addition, Na^+^, K^+^-ATPase activity is reduced in disorders of the nervous system. Na^+^, K^+^-ATPase activity was examined. The results show that Na^+^, K^+^-ATPase activity is significantly reduced in the MPTP group compared to that in the control group. Quercetin reversed the MPTP-induced reduction in Na^+^, K^+^-ATPase activity. This result indicates that quercetin protects against MPTP-induced oxidative stress and maintains the resting membrane potential of neurons.

Dopamine and acetylcholine, which play important roles in bodily movement, should maintain the dynamic equilibrium in the extrapyramidal system. Sufferers of PD experience dystonia when the cholinergic nerve is placed in an advantage status. AchE is an enzyme that degrades (via its hydrolytic activity) the neurotransmitter Ach. Acetylcholine behaves as an excitatory neurotransmitter at neuromuscular junctions [18]. In the current study, we examined the activity of AchE in the brain tissue of mice. We found that the AchE activity in the MPTP group significantly decreased compared with the control group and that quercetin reversed the MPTP-induced reduction in AchE activity. This result suggests that quercetin reduces the level of acetylcholine by enhancing the AchE activity, which may mediate quercetin-induced improvements in motor balance and coordination of MPTP-treated mice.

Dopamine is the primary neurotransmitter that is involved in motor functions. The loss of dopamine directly affects physical movements and is considered a cardinal feature of PD in humans or in animal models of the disease [19]. MPTP causes a significant reduction in brain dopamine levels. The results of our present study show that the administration of quercetin markedly improves MPTP-induced dopamine depletion in the brain tissue which is significantly altered following MPTP treatment. The enhancement of dopamine content by quercetin may restore the changes in locomotor activity.
